# No correlation between slip reduction in low-grade spondylolisthesis or change in neuroforaminal morphology and clinical outcome

**DOI:** 10.1186/1471-2474-14-245

**Published:** 2013-08-19

**Authors:** HS Femke Hagenmaier, Diyar Delawi, Nico Verschoor, F Cumhur Oner, Job LC van Susante

**Affiliations:** 1 Department of Orthopaedics, Rijnstate Hospital, Postbus 9555, Arnhem 6800 TA, The Netherlands; 2 Department of Orthopaedics, University Medical Center Utrecht, Utrecht, The Netherlands; 3 Department of Orthopaedics, Jeroen Bosch Ziekenhuis, Den Bosch, The Netherlands

**Keywords:** Slip reduction, Low-grade spondylolisthesis, Neuroforaminal morphology, Clinical outcome, Posterolateral lumbar fusion, Radiographs

## Abstract

**Background:**

In instrumented posterolateral fusion reduction of a spondylolisthesis is appealing on theoretical grounds since this may lead to indirect decompression of the entrapped nerve roots. However, there is no consensus in the literature whether a beneficial effect of reduction on outcome can be expected. The objective of the current study was to evaluate whether a correlation between the extent of listhesis reduction and clinical improvement could be established.

**Methods:**

From two ongoing prospective studies 72 patients with a single-level instrumented posterolateral lumbar fusion for low-grade spondylolisthesis (isthmic/degenerative 51/21) were evaluated. Radiographs and clinical outcome scores were available at baseline, 6 weeks and 1 year after surgery. Changes in neuroforaminal morphology were measured on calibrated radiographs. These changes in radiographic parameters were correlated to clinical outcome (Visual Analogue Score (VAS) leg pain, Oswestry Disability Index (ODI)). Fusion status was assessed on Computed Tomography-scan at one year.

**Results:**

A mean spondylolisthesis of 25 percent was reduced to 15 percent at 6 weeks with some loss of reduction to 17 percent at one year. The VAS and ODI significantly improved at both time intervals after surgery (p < 0.001). No significant correlations could be established between the extent of slip reduction and improvement in VAS or ODI (Pearson’s correlation −0.2 and 0.07 respectively at one year); this also accounted for the other radiographic parameters. A fusion rate of 64 percent was seen on CT-scan.

**Conclusions:**

Clinical outcome was not related to the obtained radiographic reduction of the slipped vertebra in patients with a lumbar fusion for low grade spondylolisthesis. Loss of reduction or non-union on CT-scans had no effect on the clinical outcome. Reduction of a low-grade spondylolisthesis in spinal fusion is appealing, however, there is no evidence that it positively affects clinical outcome on the short term.

**Trial registration:**

ISRCTN43648350

## Background

Lumbar spondylolisthesis is a common spinal disorder in adults affecting approximately 4-6% of the general population [1-6]. Slipping of the cranial vertebra generally leads to a deformation of the neuroforamen morphology with subsequent entrapment of the nerve root in the flattened and narrowed neuroforamen. Clinical presentation is variable, ranging from mild to severe symptoms of low back pain with or without sciatica [1-3,7]. The majority of patients with low-grade symptomatic spondylolisthesis can initially be treated conservatively, starting with physical therapy, activity modification and medication [1,2,4-6,8,9]. In case of persistent symptomatology, surgical treatment has proven to be superior over a conservative approach [10,11].

Various surgical techniques have been described, all aiming for decompression of the entrapped nerve roots and stabilization of the involved vertebral segment regardless of the chosen technique. Most frequently a single-level instrumented posterolateral fusion is performed [4,9,12-16].

Reduction of a spondylolisthesis is appealing on theoretical grounds since restoration of the original neuroforaminal morphology may lead to indirect decompression and restoration of the sagittal lumbosacral alignment. However, there is no consensus in the literature whether a true beneficial effect of reduction on outcome can be expected. There are arguments for and against reduction. Opponents emphasize the more extensive and expensive surgery and a higher risk of neurologic complications due to increased tension on the nerve roots during the reduction maneuver [17-20]. These arguments, however, mainly apply for the reduction of a high-grade listhesis. The extent to which this applies for the reduction of a low-grade spondylolisthesis is, however, questionable.

A review of the available literature on different surgical fusion techniques for spondylolisthesis could not declare one technique superior over the other; equally high rates of fusion and clinical improvement were described in studies with and without reduction [4,19,21]. Comparative studies on the possible beneficial clinical effect of reduction of the listhesis are difficult to find. We only found two studies that directly attempted to compare groups of patients with an instrumented spinal fusion for listhesis with and without reduction [1,8]. Both studies, however, included a limited and inhomogeneous group of patients, making it difficult to draw firm conclusions.

The aim of our study was to focus on the clinically relevant dilemma of reduction and to evaluate whether a correlation between the radiographic extent of slip reduction and clinical improvement could be established in a large group of patients with a single-level posterolateral instrumented fusion for low-grade spondylolisthesis.

## Methods

Clinical and radiographic data were derived from the databases of two ongoing prospective studies on the biological process of bony fusion in instrumented posterolateral lumbar fusion in patients with a low-grade spondylolisthesis. Consecutive radiographs were used to calculate radiographic parameters of reduction, which were subsequently correlated with the clinical outcome scores.

The first multicenter randomized controlled study evaluated the efficacy of osteogenic protein (OP-1) (Osigraft, Stryker Biotech, Hopkinton, MA) versus iliac crest autograft on the chances of spinal fusion in patients with a single-level instrumented spinal fusion for low-grade spondylolisthesis. The second study prospectively assessed bone mineral density (BMD) changes in the posterolateral fusion mass in a second group of patients with similar characteristics. All patients were treated according to a standardized surgical protocol. This consisted of a meticulous nerve root decompression (laminectomy and medial facetectomies) and posterolateral fusion using identical pedicle screw and rod instrumentation (Xia Spinal System; StrykerSpine, Allendale, NJ). Bone graft (either OP-1, iliac crest autograft or solely local bone from the laminectomy) was placed bilaterally in the thoroughly decorticated lateral gutters, alongside the instrumentation. Reduction of the listhesis was aimed for with moderate forces applied during the reduction maneuver. A pull-out of the screws was avoided at all times and no extensive release of the intervertebral disc(s) and the surrounding soft tissues was performed.

All patients were prospectively followed according to the same protocol. Radiographic images and clinical outcome scores were available preoperative, at 6 weeks and at one year follow-up. Changes in neuroforaminal morphology were measured on calibrated radiographs and subsequently correlated to clinical outcome (VAS leg pain, ODI). Fusion status was assessed on CT-scan one year after surgery. Pedicle screw positioning was thoroughly assessed at regularly taken radiographs and CT-scan at one year follow-up. Presence of any pedicular cortical breach in the medial, lateral, cranial or caudal direction was considered malpositioning. Furthermore, anterior perforation of the vertebral bodies was assessed. A perforation up to 5 millimeters through the vertebral body wall was considered acceptable.

Approval for both studies was obtained from regional ethics committees of the University Medical Center Utrecht (issue number ISRCTN43648350) and the Radboud University Nijmegen Medical Center (issue number NL 28493.091.0). Informed consent was obtained in all cases.

### Radiographic outcome measurements

To ensure adequate validation of the measurements and to minimize potential influence due to differences in magnification between the consecutive radiographs, both postoperative radiographs (6 weeks, one year) were calibrated against the length of the superior endplate on the preoperative standardized lateral radiograph. Subsequently, the remaining radiographic parameters were measured on the pre- and postoperative lateral radiographs and changes were recorded. Well-defined radiographic landmarks, as described before [22], were used to ensure adequate reproducibility of the measurements. All measurements were performed and repeated by two blinded observers (FH and JvS).

**Figure 1:**
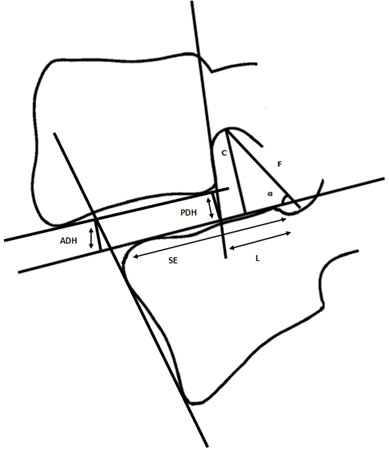
Schematic visualization of all measured radiographic parameters.

**Figure 2:**
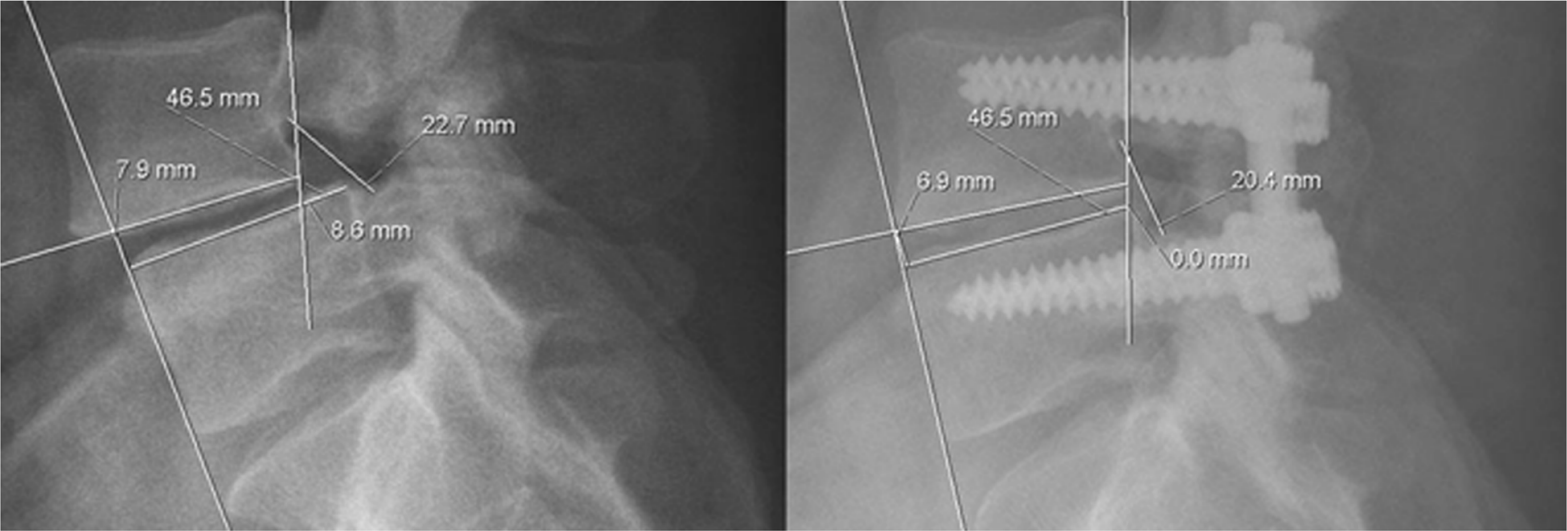
**Typical example of radiographic parameter measurements preoperative and at 1 year follow-up.** Preoperative listhesis of 8.6 mm was reduced to 0.0 mm, foraminal diameter was altered from 22.7 mm to 20.4 mm postoperatively. Anterior disc height decreased from 7.9 mm to 6.9 mm.

The following radiographic measurements were performed according to an earlier described and validated technique [22] (Figure 1, Figure 2) using the locally available PACS software package (EasyVision, Philips):

– Superior endplate (SE) (mm): diameter of the superior endplate of the inferior vertebral body of the affected segment.

– Listhesis (L): grade of listhesis measured in millimeters and percentages.

– Foraminal diameter (F) (mm): maximum distance measured from the inferior margin of the superior vertebral pedicle to the superior margin of the inferior vertebral pedicle.

– Anterior Disc Height (ADH) (mm): distance between the intersections of the vertical line drawn from the anterior surface of the inferior vertebral body with the inferior endplate of the superior vertebra and the superior endplate of the inferior vertebra, in a 90º angle with the superior endplate of the inferior vertebral body.

– Posterior Disc Height (PDH) (mm): distance between the intersections of the vertical line drawn from the posterior surface of the superior vertebral body with the superior endplate of the inferior vertebra and the inferior endplate of the superior vertebra, in a 90º angle with the superior endplate of the inferior vertebral body.

– C (mm): distance from the inferior margin of the superior vertebral pedicle, to the tangent of the extended SE-line in a 90º angle.

– α (º): measurement of listhesis in degrees, angle formed by the intersecting lines of F and the extended SE-line.

Fusion status was evaluated independently by a spinal surgeon and a radiologist on the CT-scans at one year follow-up. A third spinal surgeon was consulted if these earlier assessments were contradictory. Fusion was determined according to a modified standardized classification system based on the system previously described by Christensen et al. [23].

– Fusion: continuous bony bridge from the base of the pedicle and transverse processes from one vertebra to the other, at a minimum of one side of the spine, in absence of any secondary signs of nonunion (fracture or loosening of screws). If fusion was doubtful in any way, the patient was not classified as “fused”.

– Doubtful fusion: suboptimal quality of bone bridging or some doubtful discontinuity, including fusion mass possibly hidden behind instrumentation, at a minimum of one side of the spine, in the absence of “fusion” on the other side.

– Non-union: definite discontinuity or lack of fusion mass at both sides of the spine.

Following this classification eventually all 72 patients could be classified as having fusion “yes” or “no”, since patients where no consensus could be reached (“doubtful fusion”) were pooled with the non-union group.

### Clinical outcome measurements

A Visual Analogue Scale (VAS) [24] on leg pain on a 0–100 point scale was obtained preoperatively, at 6 weeks and one year after surgery for both legs. The highest score at baseline was considered to represent the worst affected leg; this side was used for prospective evaluation at 6 weeks and 1 year follow-up. The Oswestry Disability Index (ODI) [25] was used to evaluate subjective perception of the effect of (low) back and leg pain on quality of life*.* This is a standardized and validated questionnaire, scored from 0% (no disability) to 100% (total disability) often used to evaluate outcome in spinal pathology.

### Statistical analysis

Statistical analysis was conducted with SPSS 18.0 (SPSS Inc., Chicago, IL, 2009). Variables were controlled for normal distribution with the Kolmogorov-Smirnov test. In normally distributed data, mean, standard deviation and parametric tests (Student’s t-test) were used to analyze differences pre- and postoperatively. Associations between variables were analyzed by Pearson’s correlation coefficient. Median, range and non-parametric tests (Mann–Whitney-U and Wilcoxon-signed-rank test) were used for analysis of non-normally distributed data. Statistical significance was defined as p < 0.05.

## Results

### Study population

Seventy-two patients treated for symptomatic low-grade lumbar spondylolisthesis were included in this study, including 58 patients from the OP-1 versus iliac crest autograft study and 14 from the second prospective follow-up study. Of the first prospective (randomized) cohort 26 patients received OP-1 versus 32 patients who received iliac crest autograft. Both OP-1 and iliac crest autograft were mixed with locally obtained bone from the laminectomy. The remaining 14 patients from the second prospective follow-up study only received locally obtained bone from the laminectomy to facilitate fusion. Inclusion criteria were a low-grade lumbar spondylolisthesis (Meyerding grade I and II), degenerative or isthmic, requiring instrumented single-level posterolateral fusion and a complete radiographic and clinical follow-up, consisting of radiographic and clinical outcome measures at baseline, 6 weeks and 1 year after surgery.

The study population consisted of 33 males and 39 females, with a mean age of 51 (±12) years at time of surgery. Origin of the instability was isthmic in 51 (71%) patients and degenerative spondylolisthesis in 21 patients (29%). The most commonly affected segment was L5-S1 (49%), followed by L4-L5 (42%) and L3-L4 (9%). All demographic data is presented in Table 1.

**Table 1:** Demographic and clinical data

	**Total (N = 72)**
**Mean age at surgery (yrs)**^+^	51 (±12)
**Gender**	
Male	33
Female	39
**Level of fusion**	
L3-L4	7
L4-L5	30
L5-S1	35
**Origin of instability**	
Isthmic	51
Degenerative	21
**Meyerding classification**	
I (1–25%)	36
II (26–50%)	36
Preoperative VAS*	69.0 (2.0 – 98.0)
Preoperative ODI*	44.4 (8.9 – 73.3)

^+^ Parameters are given as the mean and (± standard deviation).

* Parameters are given as the median and (range).

### Radiographic outcome

A mean listhesis of 10.6 (±4.3) millimeters (mm) was measured preoperatively and significantly reduced to 7.2 (±4.8) mm 6 weeks after surgery (p < 0.001). After one year, in 64% of patients a mean loss of reduction of of 0.9 (±2.3) mm had occurred (p < 0.001). Foraminal diameter significantly reduced from 21.1 (±3.3) mm preoperatively to 19.3 (±4,1) and 19.0 (±4.3) at 6 weeks and one year, respectively (p < 0.001). No significant changes in anterior or posterior disc height were encountered, whereas distance C decreased from 14.3 (±3.1) preoperatively to 13.1 (±3.5) and 13.0 (±3.7).

According to the earlier described classification, in 46 patients (64%) the one year CT-scan revealed bony fusion and a non-union or doubtful fusion in 26 patients (36%). The latter consisted of 19 non-union and 7 doubtful fusion patients. All radiographic parameters are summarized in Table 2.

**Table 2:** Radiographic outcome parameters

	**Preoperative**	**Postoperative – 6 weeks**	**Postoperative – 1 year**
**SE (mm)**	42.3 (±4.5)		
**Listhesis (mm)**	10.6 (±4.3)	7.2 (±4.8)*	8.1 (±5.0)*
**Listhesis (%)**	25.2 (±10.3)	15.2 (0–54.7)*	17.4 (0–59.7)*
**F (mm)**	21.1 (±3.3)	19.3 (±4.1)*	19.0 (±4.3)*
**ADH (mm)**	7.7 (9–18.7)	8.7 (1.5-17.7)	7.6 (±3.8)
**PDH (mm)**	4.8 (±2.5)	4.8 (±2.1)	4.3 (0–10.9)
**C (mm)**	14.3 (±3.1)	13.1 (±3.5)*	13.0 (±3.7)*
**α (°)**	69.1 (±11.1)	72.5 (±11.8)*	73.9 (45.1-91.7)

Parameters are given as the mean and (± standard deviation) and median and (range), *p < 0.05.

### Clinical outcome

Both the VAS for the most affected leg and ODI improved significantly at both follow-ups after surgery (p < 0.001). The preoperative VAS for leg pain decreased from a median of 69.0 points (range 2.0-98.0) to 7.5 points (range 0.0-82.0) and 5.5 points (range 0.0-93.0) at 6 weeks and one year, respectively. The ODI improved from a preoperative score of 44.4% (range 8.9-73.3) to a median of 37.8% (range 0.0-84.4) and 11.1% (range 0.0-77.8) at 6 weeks and 1 year, respectively.

A subgroup analysis was performed on clinical outcome in patients with a “fusion” on CT-scan at one year versus patients with a “non-union”. No significant differences in improvement in VAS or ODI could be established and a similar clinical outcome was achieved for both groups. A second analysis between the isthmic versus the degenerative spondylolisthesis subgroup revealed a higher preoperative VAS for leg pain (p < 0.01) in the degenerative group. However, at both postoperative follow-ups no statistically significant difference could be established in the improvements in VAS and ODI between these two subgroups.

### Correlations

Correlation coefficients between clinical outcome (VAS/ODI) and the alteration in the various radiographic parameters at 6 weeks and one year follow-up are summarized in Table 3. Pearson’s coefficients for the correlation of slip reduction (Δ Listhesis) and the alteration of VAS leg pain after 6 weeks and one year were −0.097 and −0.204, respectively. As for the alteration of the ODI after 6 weeks and one year, the coefficients were −0.057 and 0.066. These results were not statistically significant and the relatively low coefficients indicate that no correlation could be established between slip reduction and clinical outcome. Correlation coefficients between changes in the other radiographic parameters and clinical outcome were also low and non-significantly different.

**Table 3:** Correlation coefficients for radiographic parameters and alteration of VAS leg pain and ODI at six weeks and one year follow up

	**Improvement VAS pain leg – 6 weeks**	**Improvement VAS pain leg – 1 year**	**Improvement ODI – 6 weeks**	**Improvement ODI – 1 year**
Δ **Listhesis (mm)**	−0.097	−0.204	−0.057	0.066
Δ **F (mm)**	−0.258*	−0.141	−0.018	0.120
Δ **ADH (mm)**	−0.024	0.035	0.037	0.096
Δ **PDH (mm)**	0.011	0.230	−0.017	0.103
Δ **C (mm)**	−0.225	−0.185	−0.050	−0.039
Δ **α (mm)**	−0.123	0.061	−0.083	−0.060

Data are given as Pearson’s correlation coefficients, * *p* < 0.05.

**Figure 3:**
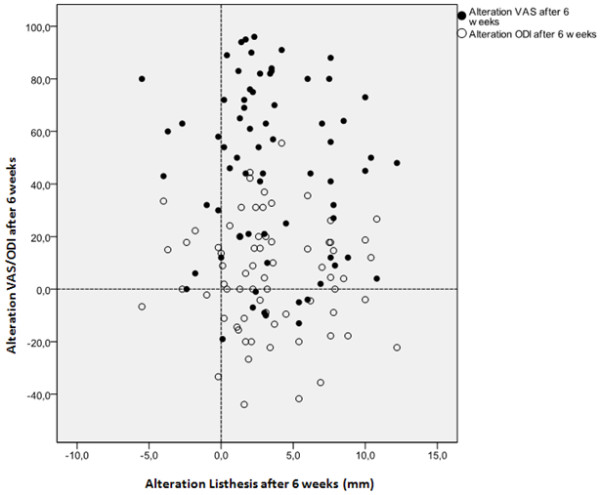
**Visualization of the correlation between the alteration of listhesis and clinical outcome at 6 weeks follow-up, demonstrating clinical improvement and radiographic reduction.** Random distribution indicates that there is no correlation.

**Figure 4:**
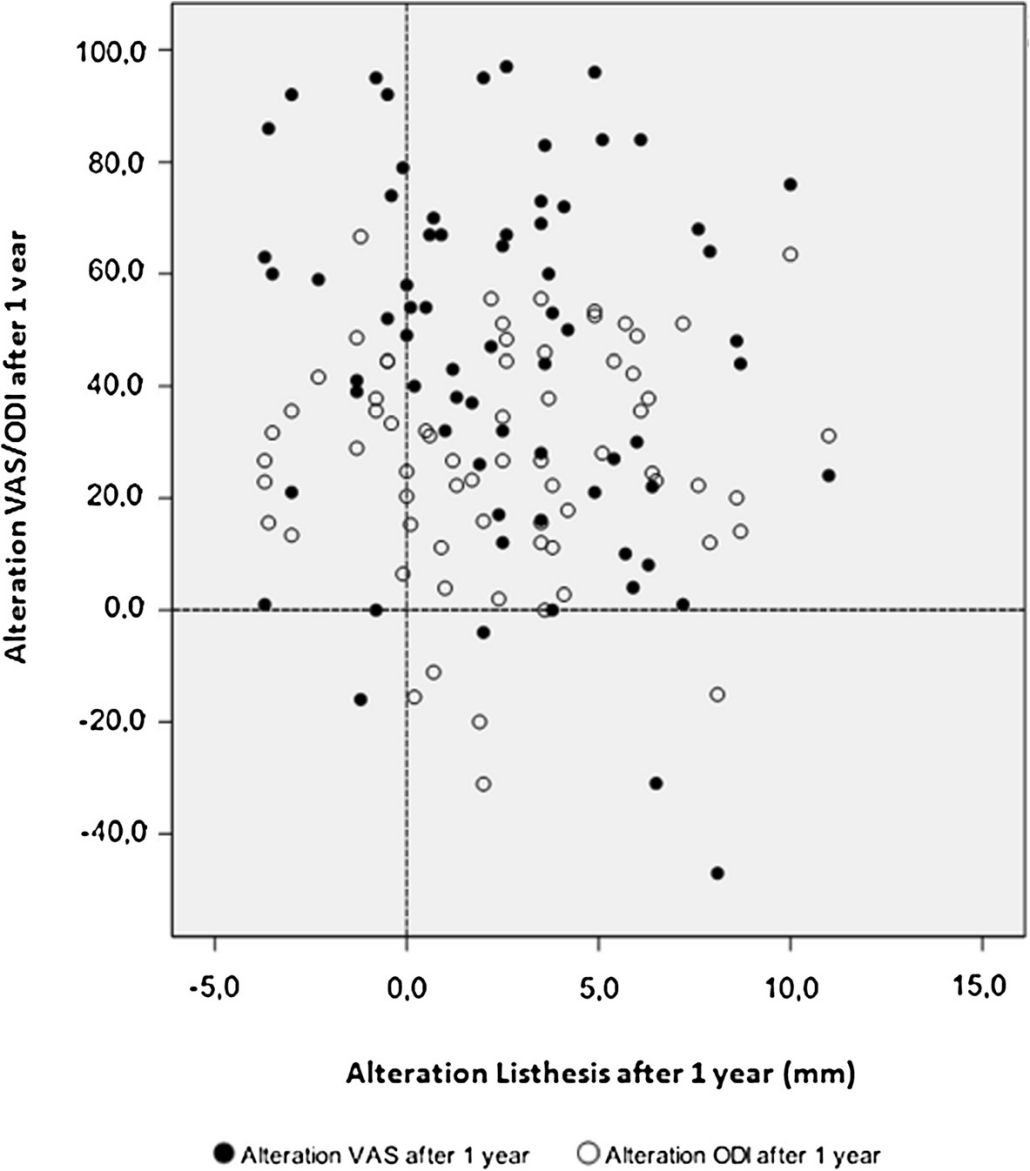
**Visualization of the correlation between the alteration of listhesis and clinical outcome at 1 year follow-up, demonstrating some loss of reduction and further clinical improvement.** Random distribution indicates that there is no correlation.

The scatterplots at 6 weeks (Figure 3) and one year (Figure 4) represent the correlation coefficients for each patient between changes in VAS and ODI versus changes in slip reduction (Δ Listhesis). As one would expect the majority of patients is situated in the upper-right quadrant of the scatterplot, indicating clinical improvement and radiographic reduction. However, distribution of the plots is rather inhomogeneous and no correlation could be established. In the 6-weeks scatterplot there is still a substantial number of patients with a negative change in especially the ODI score, whereas in the one-year scatterplot almost all patients revealed an improvement in their clinical outcome.

## Discussion

In this study we were not able to establish a correlation between the amount of radiographic reduction of the slipped vertebra and clinical improvement of leg and back pain in patients treated with instrumented lumbar fusion for symptomatic low-grade spondylolisthesis. Although we were able to reduce the listhesis to some extent in 85% of patients at 6 weeks follow-up, some loss of reduction was commonly encountered. Nonetheless, clinical improvement was very satisfactory with a highly significant decrease in VAS leg pain and ODI at final follow-up, which is in accordance with two earlier reports [2,4]. In these studies, a mean loss of reduction of 5-6% was reported after instrumented fusion, without a statistically significant effect on good clinical outcome.

Despite the fact that reduction of a spondylolisthesis is commonly advocated there are only two studies that have reported on a possible relation between reduction itself and improvement of clinical outcome. The first study by Benli et al. [8] prospectively compared patients with and without reduction as well as high- and low-grade spondylolisthesis who underwent a posterior instrumented fusion. After a mean follow-up of 38 months no statistically significant differences in clinical outcome could be established between groups with or without reduction. Furthermore, no significant differences were encountered between the low- and high-grade dysplastic spondylolisthesis groups. Although this study generated interesting data, it remains difficult to draw firm conclusions due to the limited number of patients included in the 4 subgroups, comprising both high- and low-grade slips. The second study by Audat et al. [1] compared posterior lumbar interbody fusion (PLIF) with reduction versus fusion in-situ in 41 patients with symptomatic low-grade (Meyerding I and II) spondylolisthesis. At latest follow-up again both groups showed no influence of reduction on clinical outcome in a limited number of patients. To our knowledge, our study is the first to correlate clinical outcome score improvement to the extent of radiographic reduction of the spondylolisthesis in a substantial number of patients.

Strengths of the present study are reflected in the relative homogeneity as well as the size (N = 72) of our study population. Only low-grade spondylolisthesis patients were included and all patients were operated according to a well-defined instrumented spinal fusion protocol [23], using identical pedicle screw and rod instrumentation. There are, however, some limitations to our study. First, the selected population was not truly homogeneous, consisting of both isthmic and degenerative origin of the spondylolisthesis. Furthermore, not all patients could be classified as “fused” on CT-scan one year after surgery. However, we do not believe that these variables have accounted for major confounding factors, since our subgroup analysis regarding patients with an isthmic spondylolisthesis and “fusion” on CT-scan did not show significant differences in improvement of clinical outcome compared to the entire group of 72 patients. The scatterplot for this subgroup of patients (N = 36) revealed an equal distribution of plots with similarly low correlation coefficients. These results are consistent with earlier reports from the literature, suggesting that presence or absence of a true bony fusion does not influence clinical improvement of patients after lumbar fusion surgery for either isthmic or degenerative spondylolisthesis, certainly not at early follow-up [4,13,19,26-30].

Secondly, all radiographic parameters were measured on plain lateral radiographs of the lumbar spine taken at three consecutive intervals. To minimize a possible influence of a difference in magnification between those radiographs, we have calibrated both postoperative radiographs against the length of the superior endplate as measured on the preoperative radiograph. This way the reported radiographic distance measurements on the consecutive radiographs appeared to be validated.

Lastly, the use of bone morphogenetic proteins can be a confounding factor. Concerns were addressed regarding complications associated with the use of BMP-2 in spinal indications, which include radiculitis, osteolysis, retrograde ejaculation, and ectopic bone formation [31]. For OP-1, as used in the current study, no major complications were reported associated with its use, although few case reports were published on ectopic bone formation [32,33]. Additionally, no product related adverse events occurred in our study comparing OP-1 with iliac crest autograft. Therefore, the usage of BMPs in a subgroup of our study population is not expected to be of a major influence on the conclusions of this study.

In conclusion, we feel that with this study we were able to gain further insight in the clinical dilemma whether reduction of a low-grade spondylolisthesis should be an important aim in the surgical treatment of spondylolisthesis. The reduction maneuver remains appealing, yet on theoretical grounds. Currently there is no consensus or conclusive evidence that it positively affects clinical outcome. The results from our study offer the best available evidence to date that there appears to be no correlation between the amount of reduction of a spondylolisthesis and the improvement in leg and back pain after instrumented posterolateral spinal fusion.

## Conclusions

• The objective of this study was to evaluate whether a correlation between the extent of slip reduction and clinical improvement could be established after a single level instrumented posterolateral spinal fusion for low-grade spondylolisthesis.

• The mean preoperative spondylolisthesis of 25 percent was reduced to 15 percent at 6 weeks with some loss of reduction to 17 percent at one year. Both the VAS for leg pain and ODI significantly improved at both time intervals after surgery (p < 0.001).

• Clinical outcome was not related to the obtained radiographic reduction of the slipped vertebra. Although reduction remains appealing, there is no evidence that it positively affects clinical outcome.

## Abbreviations

VAS: Visual analogue scale; ODI: Oswestry disability index; CT-scan: Computed tomography-scan; OP-1: Osteogenic protein-1; BMD: Bone mineral density.

## Competing interests

The authors declare that they have no competing interests.

## Authors’ contributions

FH: Data collection, measurements, statistical analysis, drafting of the manuscript. DD: Data collection, participation in study design and coordination, critically revising manuscript for important intellectual content and interpretation of data. NV: Data collection, participation in study design and coordination, critically revising manuscript for important intellectual content and interpretation of data. FCO: Participation in study design and coordination, critically revising manuscript for important intellectual content and interpretation of data. JvS: Conceived of the study, design and coordination, drafting of the manuscript, critically revising manuscript for important intellectual content and interpretation of data. All authors have read and approved the final manuscript.

## Pre-publication history

The pre-publication history for this paper can be accessed here:

http://www.biomedcentral.com/1471-2474/14/245/prepub
